# The novel role of T2R14 antagonism, by LW129, on VEGF‐induced lung endothelial neovascularisation processes in vitro

**DOI:** 10.14814/phy2.71168

**Published:** 2026-10-30

**Authors:** Lewis Spurrier‐Best, Ellizabeth O. Harrington, Havovi Chichger

**Affiliations:** ^1^ Biomedical and Health Innovation Research Centre Anglia Ruskin University Cambridge UK; ^2^ Vascular Research Lab, Providence Veterans Affairs Medical Center Brown University Providence Rhode Island USA

**Keywords:** angiogenesis, bitter taste receptors, endothelial function, lung microvasculature, T2R14, VEGF

## Abstract

Pulmonary vascular remodelling, driven by dysregulated angiogenesis, is a hallmark of several cardiopulmonary diseases, yet the molecular pathways regulating endothelial responses remain incompletely understood. Bitter taste receptors (T2Rs), traditionally recognised as chemosensory receptors, have emerged as important modulators of vascular function. However, the contribution of T2Rs to pulmonary angiogenesis remains unexplored. Here, we investigated the role of T2R14 in vascular endothelial growth factor (VEGF)‐mediated angiogenic responses in human pulmonary microvascular endothelial cells (HPMECs). Using the selective T2R14 antagonist LW129, we examined the effects of T2R14 inhibition on key endothelial angiogenic processes, including VEGF‐induced proliferation, migration and capillary‐like tube formation in vitro. Pharmacological antagonism of T2R14 significantly attenuated VEGF‐driven endothelial proliferative, migratory and morphogenic responses, revealing a novel contribution of T2R14 signalling to pulmonary microvascular angiogenesis. Proteome Profiler analysis identified a range of molecular regulators of angiogenic processes which were dysregulated by LW129 and VEGF exposure, suggesting a potential role for CXCL16, endoglin, FGF‐7, and VEGF‐C. These findings identify T2R14 as a novel regulator of VEGF‐dependent endothelial activation and suggest that targeting T2R14 signalling may represent a therapeutic strategy to limit pathological pulmonary vascular remodelling associated with aberrant angiogenesis.

## 1 INTRODUCTION

Angiogenesis, the process by which new blood vessels arise from pre‐existing vasculature, is a tightly regulated physiological mechanism essential for embryonic development, tissue repair and maintenance of organ homeostasis. Dysregulated angiogenesis contributes to numerous pathological conditions, including cancer, chronic inflammatory disorders, pulmonary hypertension and fibrotic lung diseases (Ackermann et al., 2024). Endothelial cells are the principal cellular mediators of angiogenesis, coordinating vessel sprouting, migration, proliferation and lumen formation in response to environmental and molecular cues (Eelen et al., 2020). Among the endothelial populations within the lung, pulmonary microvascular endothelial cells (HPMECs) are particularly important as they maintain alveolar‐capillary integrity and regulate vascular remodelling during health and disease (Ackermann et al., 2024; Cao et al., 2016).

Vascular endothelial growth factor A (VEGF‐A) is widely recognised as the central regulator of angiogenesis. Binding of VEGF‐A to vascular endothelial growth factor receptor‐2 (VEGFR2) initiates receptor dimerisation and phosphorylation, triggering downstream signalling pathways including phosphoinositide 3‐kinase (PI3K)/Akt, phospholipase C‐γ (PLCγ), extracellular signal‐regulated kinase (ERK) and focal adhesion kinase (FAK) (Song & Finley, 2022; Sun et al., 2012). Activation of these pathways promotes endothelial cell proliferation, migration, survival and capillary network formation, all of which are hallmarks of angiogenic remodelling (Fujio & Walsh, 1999; Gerber et al., 1998; Perez‐Gutierrez & Ferrara, 2023). The VEGF–VEGFR2 axis is therefore considered a master regulator of both physiological and pathological neovascularisation and remains a major therapeutic target in vascular disease and cancer (Dudley & Griffioen, 2023; Shibuya, 2011). Within the pulmonary circulation, VEGF signalling plays a complex role. Appropriate VEGF activity is required for lung development, endothelial survival and maintenance of alveolar architecture; however, excessive or aberrant VEGF signalling can contribute to pathological vascular remodelling, increased permeability and disease progression (Ourradi et al., 2017; Perez‐Gutierrez & Ferrara, 2023). Altered VEGF expression and signalling have been implicated in acute lung injury, pulmonary hypertension, pulmonary fibrosis and lung cancer, highlighting the need to better understand modulators of endothelial VEGF responses in the pulmonary microvasculature (Lu et al., 2021; Ourradi et al., 2017; Papaioannou et al., 2006; Walsh et al., 2025). Recent studies using pulmonary endothelial models have further demonstrated that VEGF‐driven migration and network formation are tightly regulated by multiple endogenous signalling pathways, demonstrating the complexity of angiogenic control within the pulmonary vasculature (Becker et al., 2001; Mirza et al., 2024; Stevens et al., 2022). Therefore, understanding the molecular mechanisms underlying VEGF‐mediated control of the pulmonary vasculature has significant therapeutic potential.

Bitter taste receptors (T2Rs) are G‐protein‐coupled receptors which were initially identified in gustatory tissues but are now recognised as broadly expressed in extraoral organs, including the respiratory system, immune cells, smooth muscle and vascular endothelium (Chandrashekar et al., 2000; Foster et al., 2013; Kertesz et al., 2022). Increasing evidence suggests that these receptors function as chemosensory regulators of cellular physiology beyond taste perception. Activation of T2Rs can influence intracellular calcium signalling, cyclic nucleotide production, nitric oxide generation, inflammatory responses and cellular barrier function (Carey et al., 2017; McMahon et al., 2022; Robinett et al., 2014), suggesting a broader role in vascular biology and tissue homeostasis. Among the approximately 25 human T2R subtypes, T2R14 is one of the most broadly tuned receptors, capable of recognising a diverse range of structurally distinct ligands (Fierro et al., 2023; Karaman et al., 2016; Nowak et al., 2018). T2R14 is expressed throughout the respiratory tract, including airway smooth muscle, epithelial cells and pulmonary endothelial cells (Foster et al., 2013; Kertesz et al., 2022; Robinett et al., 2014). Importantly, our previous studies show that pulmonary endothelial cells exhibit relatively high expression of T2R14 compared with several other T2R family members, suggesting a key role for T2R14 in the regulation of endothelial function (Kertesz et al., 2022). We further demonstrated that T2R14 activation exacerbates LPS‐induced barrier dysfunction in pulmonary microvascular endothelial cells, a finding which contrasted with the other highly expressed T2Rs in the lung endothelium, T2R10 and T2R38. Whilst this indicates a receptor‐specific function of T2R14 within the pulmonary vasculature, the role of T2R14 in angiogenic signalling remains poorly defined. Despite growing interest in extraoral bitter taste receptors as therapeutic targets, to date, there are no studies investigating the role of T2Rs in regulating angiogenic processes or VEGF signalling. Given the central role of VEGF in driving endothelial migration, proliferation and tube formation, elucidating whether T2R14 signalling intersects with canonical angiogenic pathways may reveal previously unrecognised mechanisms regulating pulmonary vascular remodelling. Furthermore, understanding the consequences of pharmacological inhibition of T2R14 could provide insight into the therapeutic potential of targeting this receptor in diseases characterised by aberrant angiogenesis.

The present study investigated the role of T2R14 in VEGF‐induced angiogenic responses in pulmonary microvascular endothelial cells. Using a selective pharmacological antagonist of T2R14, LW129, we examined the role of T2R14 on key angiogenic processes, including endothelial proliferation, migration and capillary‐like tube formation in vitro. Our findings identify a previously unrecognised role for T2R14 signalling in promoting VEGF‐driven angiogenesis and demonstrate that T2R14 antagonism attenuates angiogenic activity in HPMECs, highlighting this receptor as a potential therapeutic target for pathological pulmonary vascular remodelling.

## 2 RESULTS

### 2.1 Toxic effects of T2R14 agonists and antagonist in lung microvascular endothelial cells

As a promiscuous T2R, agonists for T2R14 belong to a structurally diverse group of chemicals including flavones, microbial metabolites and developed drugs such as antihistamines and NSAIDs (Di Pizio et al., 2020; Karaman et al., 2016; Levit et al., 2014). In contrast, there are far fewer studies investigating antagonists for T2R14. In 2023, 10 potential antagonists were identified through virtual screening (Fierro et al., 2023) and, of these antagonists, the compound with the lowest IC_50_ in T2R14‐transfected HEK cells when tested against the agonist flufenamic acid was LW129. Therefore, cell viability in HPMECs was first investigated to establish the impact of a range of T2R14 agonists, flufenamic acid (Figure 1a), aristolochic acid (Figure 1b), nobiletin (Figure 1c), luteolin (Figure 1d), tangeretin (Figure 1e) and noscapine (Figure 1f), and the antagonist, LW129 (Figure 1g). All agonists had no significant impact on endothelial cell viability up to 10 μM, after which decreased viability was noted (Figure 1a–d). However, at concentrations of 100 μM, tangeretin (Figure 1e) and noscapine (Figure 1f) caused a significant decrease in viability. In contrast to the agonists, the antagonist LW129 significantly increased cell viability at concentrations from 1 nM to 10 μM, with a significant decrease in cell viability only noted at 100 μM (Figure 1g). These findings show that LW129 increases endothelial cell viability at concentrations significantly lower than the reported IC_50_, whereas T2R14 agonists do not significantly impact endothelial cell viability at lower concentrations.

**FIGURE 1:**
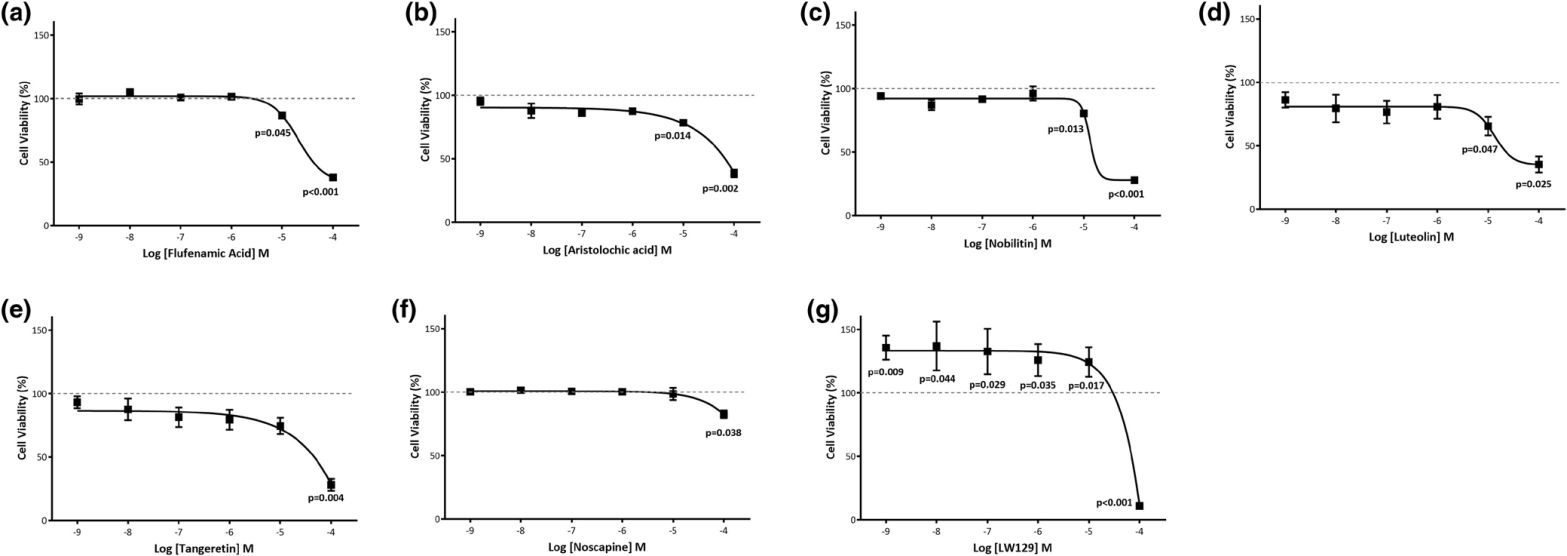
Toxic effects of T2R14 agonists and antagonist on lung microvascular endothelial cells. T2R14 agonists, flufenamic acid (a), aristolochic acid (b), nobilitin (c), luteolin (d), tangeretin (e), noscapine (f) and T2R14 antagonist, LW129 (g) impact on endothelial cell viability was measured using MTT assay following 24 h exposure to relevant compound. Data are normalized to vehicle treatment (100%) which is denoted by dashed line. Data are presented as mean ± S.E.M. *n* = 3–4. *p* values on graph at relevant points versus vehicle for agonist or antagonist (100%).

### 2.2 T2R14 antagonist, LW129, impacts baseline endothelial cell proliferation and migration

Given the effect of the T2R14 antagonist, LW129, on HPMEC viability, the next studies investigated endothelial cell proliferation at 500 nM; a concentration where increased cell viability was observed but where antagonist effect on T2R14 has previously been observed (Fierro et al., 2023). Following 24 h exposure with LW129, endothelial cell proliferation was significantly increased from 100% ± 7.65% with vehicle control to 144.88% ± 8.27% with LW129 (Figure 2a). Similarly, endothelial cell migration was significantly increased from 100% ± 5.09% with vehicle control to 123.73% ± 2.99% with LW129 exposure (Figure 2b). In contrast, HPMEC adhesion (Figure 2c), tube formation (Figure 2d), and endothelial cell VEGF release (Figure 2e) were not significantly impacted by LW129 exposure. These findings suggest a pro‐proliferative and pro‐migratory endothelial cell phenotype following exposure to the T2R14 antagonist.

**FIGURE 2:**
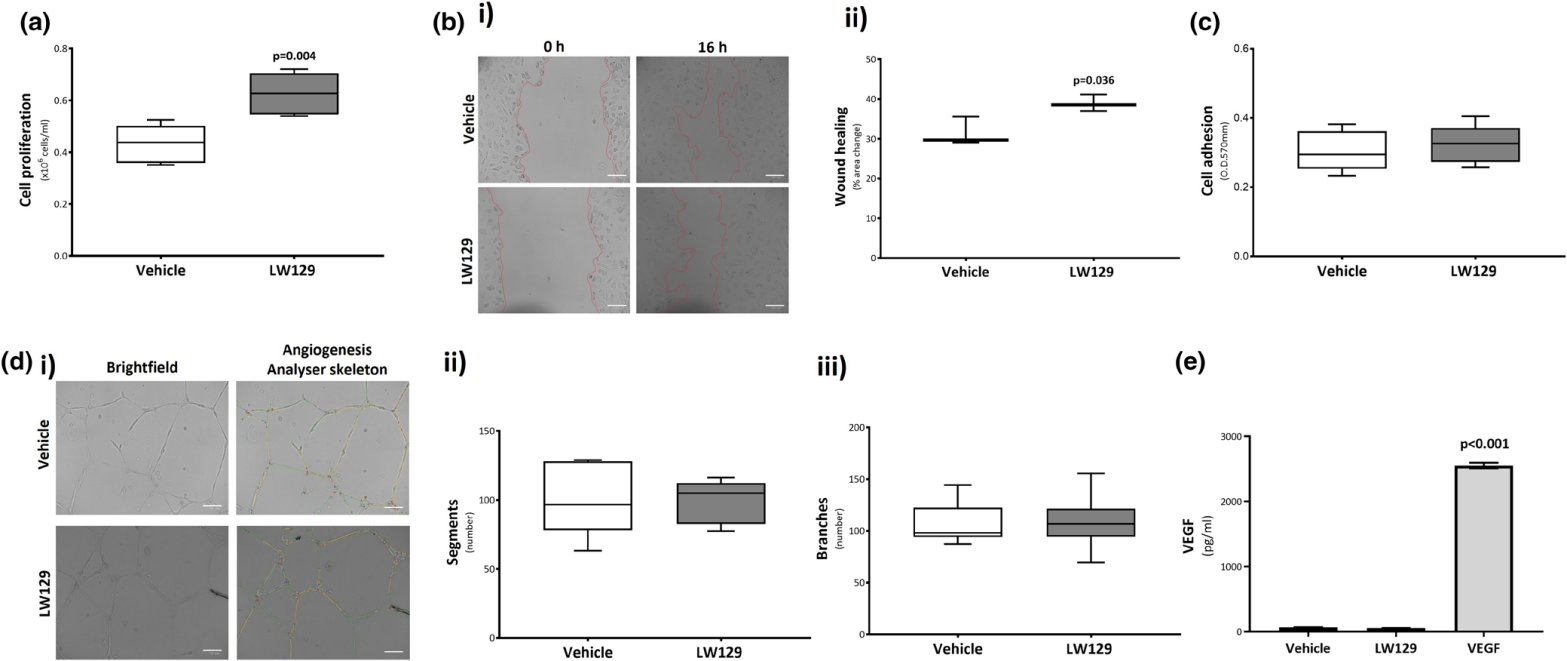
T2R14 antagonist, LW129, increases cell proliferation and migration, but not adhesion or angiogenic potential, and has no impact on baseline VEGF levels. Endothelial cell proliferation (a) was measured with cell count following 24 h exposure to LW129 (500 nM). Cell migration (b) was measured by scratch assay at 16 h post‐scratch and post‐treatment (b) with representative image (i) and quantification (ii) included, whereas cell adhesion was measured at 6 h post plating and treatment (c). Angiogenic potential was assessed using HPMEC plated in Matrigel™ and following 24 h exposure to LW129 (d) with representative image (i), and quantification of segment (ii) and branch (iii) number included. VEGF expression was measured using ELISA of lysates from HPMECs exposed to LW129 (500 nM) or VEGF (100 ng/mL) (e). Scale bar on representative images (b i and d i) of 100 μm and box and whisker plots present median and minimum and maximum data point as lower and upper error bar. Data are presented as mean ± S.E.M. *n* = 3–6. *p* values on graph at relevant points.

### 2.3 T2R14 antagonist, LW129, attenuates VEGF‐induced angiogenic processes in the pulmonary endothelium

We next sought to understand the effect of LW129 on in vitro angiogenic processes in the pulmonary endothelium. Whilst endothelial cell proliferation was significantly elevated by LW129 exposure alone, VEGF‐induced proliferation was attenuated by co‐treatment with LW129 (Figure 3a). Similarly, VEGF‐induced adhesion (Figure 3b), migration (Figure 3c) and tube formation (Figure 3d) were blunted by co‐exposure with LW129. Taken together, these data demonstrate a key role for T2R14 in regulating angiogenic processes in the pulmonary microvasculature.

**FIGURE 3:**
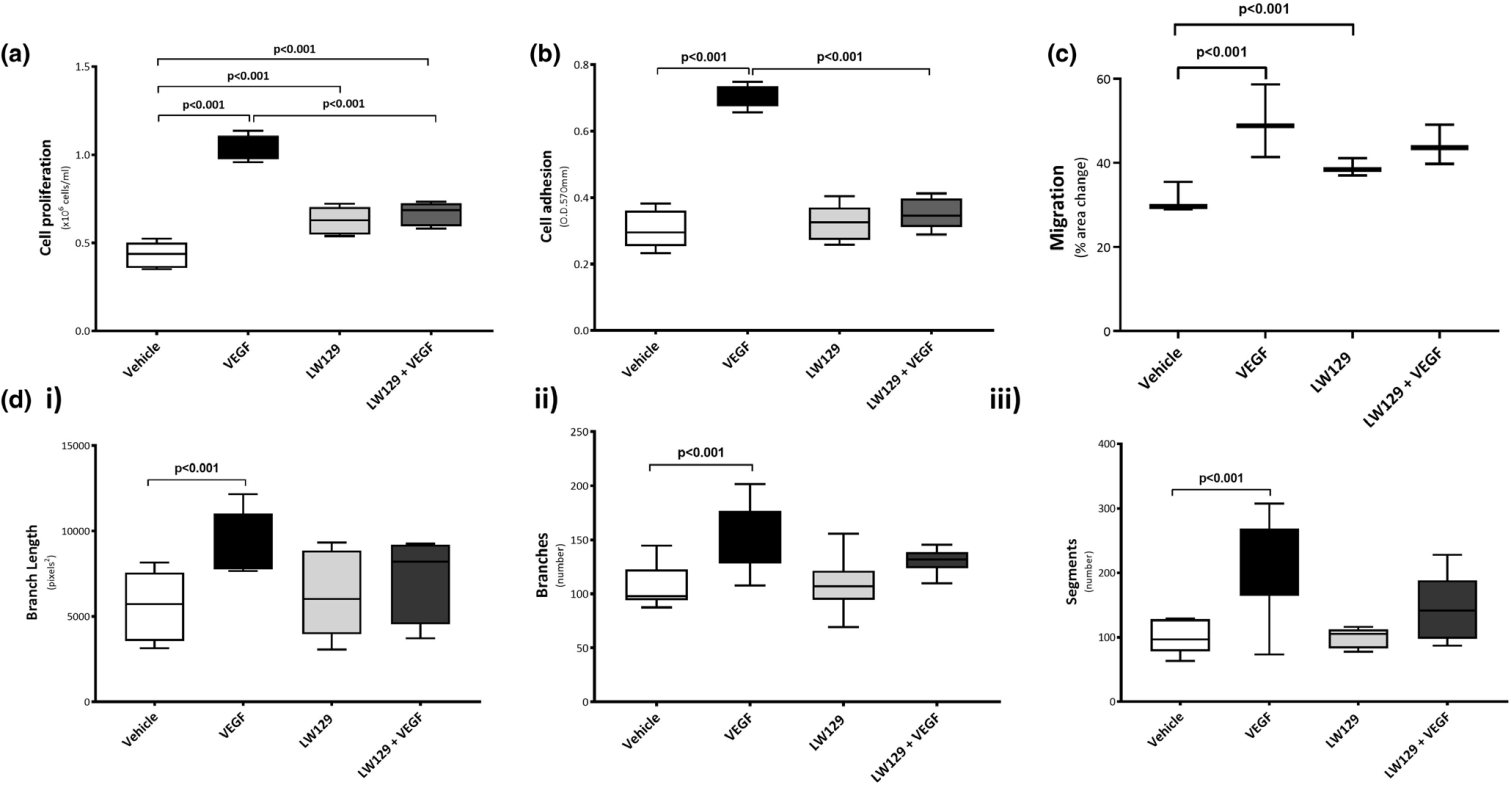
T2R14 antagonist attenuates VEGF‐induced cell proliferation, adhesion, migration and angiogenic potential. Endothelial cell proliferation (a), adhesion (b), migration (c) and angiogenic processes (d) were measured in HPMECs exposed to LW129 (500 nM) in the presence and absence of VEGF (100 ng/mL). Box and whisker plots present median and minimum and maximum data point as lower and upper error bar. Data are presented as mean ± S.E.M. *n* = 3–6. *p* values on graph at relevant points.

### 2.4 LW129 regulates expression of key angiogenic proteins in the lung endothelium in vitro

To further understand how LW129 regulates VEGF‐induced angiogenic processes, our final experiments sought to investigate the molecular changes in HPMEC following exposure to the antagonist in the presence and absence of VEGF. Using the Proteome Profiler™ Human Angiogenesis array, we screened cell lysates for changes in expression of angiogenesis‐specific regulators. Efficacy of the assay was confirmed by significantly increased VEGF‐A expression noted following exposure to VEGF with 31.82 ± 0.84‐fold increase with VEGF treatment alone (*p* < 0.05) and 31.35 ± 0.42‐fold increase with LW129 + VEGF treatment (*p* < 0.05) compared to vehicle. Of note, and in agreement with Figure 2e, LW129 exposure alone did not significantly impact VEGF‐A expression (1.46 ± 0.84‐fold compared to vehicle, *p* > 0.05) (Figure 4a i). Indeed, although a change in expression of a range of angiogenesis regulators was noted following exposure to LW129, only CXCL16 and PDGF‐AB/PDGF‐BB levels were significantly upregulated with the T2R14 antagonist (Figure 4a ii). In contrast, expression of a range of the angiogenesis regulators was impacted by VEGF in the presence and absence of LW129 (Figure 4b). Significant increased expression in CXCL16 following LW129 exposure was attenuated with combined VEGF and LW129 treatment (Figure 4c i). There was also a small but significant decrease in endoglin (Figure 4c ii), FGF‐7 (Figure 4c iii), and VEGF‐C (Figure 4c iv) expression following exposure to both VEGF and LW129 as compared to VEGF treatment only. Interestingly, VEGF‐induced increase in VEGF‐C expression was attenuated by co‐treatment with LW129 (Figure 4c iv). Taken together, these findings indicate a potential molecular mechanism through which LW129 could regulate angiogenic processes in the lung endothelium in vitro.

**FIGURE 4:**
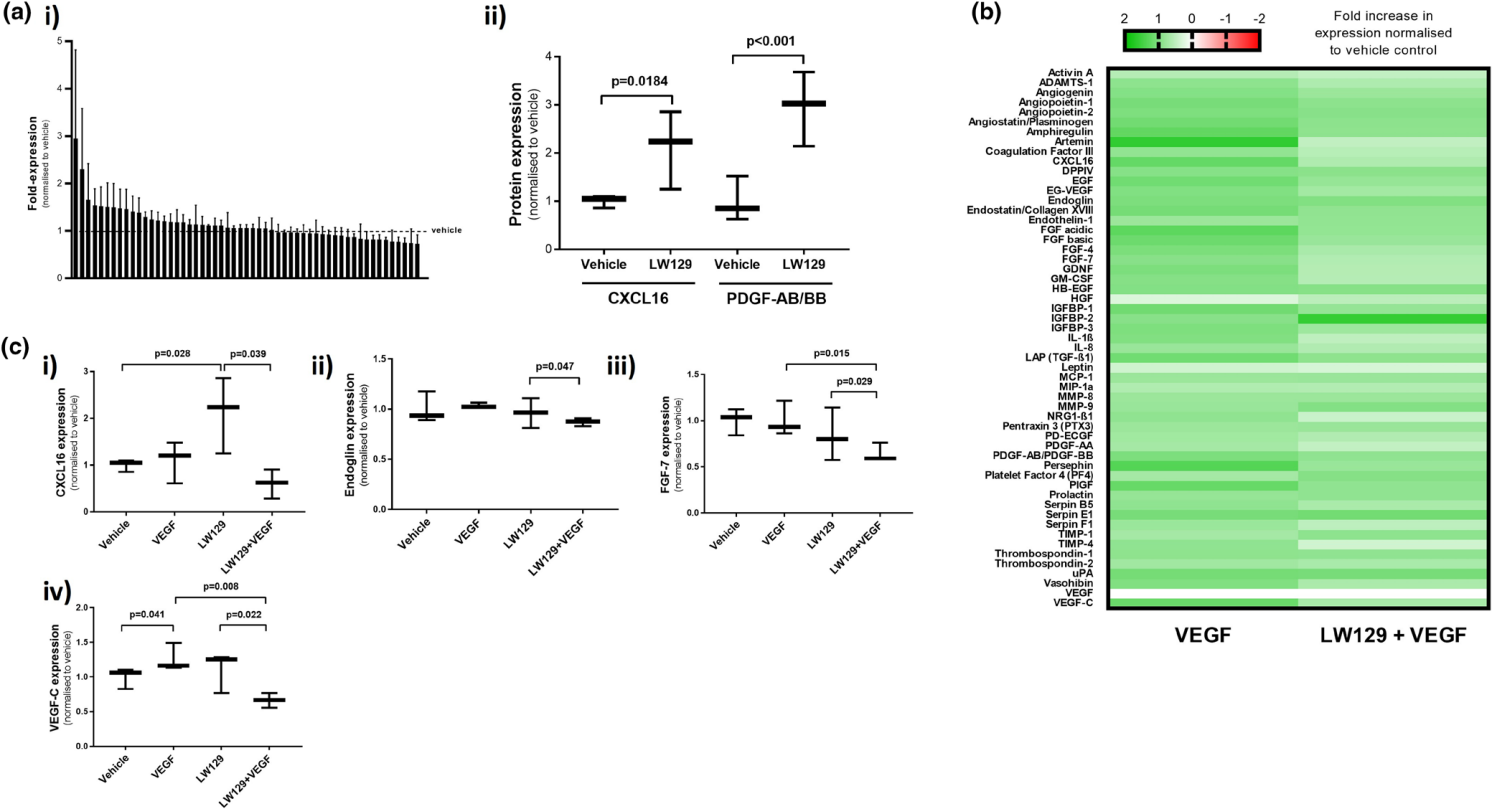
T2R14 antagonist exposure causes significant upregulation of CXCL16 and PDGF‐AA/BB, and, with VEGF co‐treatment, reduces expression of CXCL16, endoglin, FGF‐7 and VEGF‐C. HPMECs were exposed to LW129 (500 nM) in the presence and absence of VEGF (100 ng/mL). Cell lysates were collected and used for Proteome Profiler Angiogenesis assay membranes. Changes in the expression of angiogenesis‐related proteins were normalized to vehicle and presented as fold‐expression with the effect of VEGF alone (a, i) or LW129 alone (a, ii) demonstrated as volcano plot. Changes in expression with LW129 in the presence and absence of VEGF, as normalized to vehicle, is shown as a heat map (b). Specific changes in expression were analysed (c) for CXCL16 (i), endoglin (ii), FGF‐7 (iii) and VEGF‐C (iv). Dashed line represents vehicle normalization (a). Data are presented as mean ± S.E.M. *n* = 3. *p* values on graph at relevant points.

## 3 DISCUSSION

Angiogenesis is a fundamental driver of pulmonary vascular remodelling in numerous cardiopulmonary diseases, yet the molecular pathways that modulate endothelial responses to pro‐angiogenic stimuli remain incompletely understood. In the present study, we identify the bitter taste receptor T2R14 as a novel regulator of VEGF‐induced angiogenic responses in the pulmonary microvasculature. Pharmacological inhibition of T2R14, with the selective antagonist LW129, significantly attenuated endothelial proliferation, migration and capillary‐like tube formation, demonstrating that T2R14 signalling facilitates multiple key steps of the angiogenic process. These findings extend the functional repertoire of extraoral bitter taste receptors beyond their established chemosensory and innate immune roles and implicate T2R14 as a novel modulator of pulmonary endothelial biology with potential relevance to pathological pulmonary vascular remodelling.

Pulmonary vascular remodelling is a hallmark of numerous cardiopulmonary disorders, including pulmonary arterial hypertension, chronic obstructive pulmonary disease, and interstitial lung disease, and is the principal pathological process underlying progressive increases in pulmonary vascular resistance and right ventricular dysfunction (Ghofrani et al., 2025; Mocumbi et al., 2024). Increasing evidence suggests that endothelial dysfunction represents one of the earliest initiating events in vascular remodelling, promoting a phenotypic shift from a quiescent, vasoprotective endothelium towards a proliferative, pro‐inflammatory and apoptosis‐resistant state that drives pathological angiogenesis and vascular remodelling. Among the signalling pathways governing endothelial function, VEGF signalling is recognised as the central regulator of angiogenesis. Persistent or dysregulated VEGF signalling promotes pathological angiogenesis characterised by excessive endothelial proliferation, aberrant vessel architecture, and increased vascular permeability, processes that are increasingly recognised as important contributors to pulmonary vascular remodelling (Perez‐Gutierrez & Ferrara, 2023). Indeed, in the present study, we utilised VEGF as a pro‐angiogenic insult to assess the effect of LW129. As expected from the literature, exposure of endothelial cells to VEGF increased a range of angiogenic processes investigated, including proliferation and capillary‐like tube formation. Our studies with the Angiogenesis Proteome Profiler™ indicated that, unsurprisingly, VEGF exposure significantly increased VEGF‐A and VEGF‐C expression. Interestingly, VEGF exposure had no effect on the protein expression of some key angiogenic regulators, such as CXCL16, FGF‐7, and endoglin. Although VEGF is a central regulator of endothelial proliferation, survival, migration and vascular permeability, its downstream effects are highly dependent on cell type, exposure duration and the presence of co‐stimulatory signals such as inflammation, hypoxia, or mechanical stress, which modulate the endothelial response (Perez‐Gutierrez & Ferrara, 2023). For example, CXCL16 is typically upregulated following activation of inflammatory pathways, while PDGF‐AA/PDGF‐BB expression is often associated with vascular maturation and tissue remodelling rather than acute VEGF signalling (Abel et al., 2004; Isozaki et al., 2013). Furthermore, VEGF‐induced signalling is highly dynamic and may predominantly influence gene transcription, protein phosphorylation, secretion, or receptor activation rather than steady‐state intracellular protein levels (Abhinand et al., 2023; Kim et al., 2007). This supports the growing appreciation that pulmonary vascular remodelling results from dysregulated signalling networks, rather than isolated molecular abnormalities. This has generated considerable interest in identifying novel modulators that fine‐tune endothelial responsiveness to VEGF. Such regulators may offer opportunities to selectively attenuate pathological angiogenesis while preserving physiological endothelial repair, representing an attractive therapeutic strategy for pulmonary vascular disease.

In the present study, we identified a novel anti‐VEGF approach which attenuates VEGF‐induced angiogenic processes. We have previously demonstrated the presence of T2R14 in the pulmonary endothelium, and that activation of the receptor increases endothelial barrier dysfunction in vitro; however, there are no current studies linking the bitter taste receptor to angiogenic processes (Kertesz et al., 2022). Given that T2R14 has been implicated in regulating cellular responses to environmental and endogenous ligands, the pharmacological antagonist was utilised in the present study to investigate how T2R14 impacts angiogenic processes. In heterologous expression systems, LW129 inhibited T2R14‐mediated signalling induced by agonists such as flufenamic acid, demonstrating its utility for dissecting receptor‐specific responses (Fierro et al., 2023; Levit et al., 2014). Interestingly, LW129 did not markedly affect adhesion or tube formation in the lung microvascular endothelial cells but did cause a small increase in cell proliferation and migration, potentially through elevated protein expression of PDGF‐AA/BB or CXCL‐16. PDGF‐AA/BB is a potent regulator of endothelial behaviour, promoting endothelial proliferation through PDGF receptor signalling, particularly via PDGFR‐β activation and downstream MAPK/PI3K pathways (Battegay et al., 1994; Thommen et al., 1997). Similarly, soluble CXCL16 has been shown to stimulate endothelial cell migration and proliferation via ERK1/2, p38 MAPK and PI3K–AKT signalling and induction of HIF‐1α and VEGF in endothelial cells (Isozaki et al., 2013; Zhuge et al., 2005). However, it is worth noting that LW129 did not have any impact on baseline endothelial cell adhesion or capillary‐like tube formation, suggesting a proliferation‐ and migration‐specific effect which could impact normal endothelial cell function.

A key finding from the present study is that LW129 caused reduction of VEGF‐induced migration and proliferation, and attenuation of VEGF‐induced adhesion and tube formation. Whilst these studies use primary human endothelial cells, rather than showing the effect in an in vivo model, this suppression of VEGF‐induced angiogenic processes suggests a key role for T2R14 in regulating these processes in the pulmonary endothelium. These findings suggest that T2R14 signalling may differentially regulate endothelial cell behaviour depending on the cellular and angiogenic context, rather than acting as a universal positive regulator of angiogenesis. The increased basal proliferation and migration observed following LW129 treatment, alongside its lack of effect on endothelial adhesion or tube formation, further support a context‐dependent role for T2R14 in regulating distinct aspects of endothelial function. Fierro et al. demonstrated the specificity of LW129 using the response of T2R14‐overexpressing HEK cells to the agonist, flufenamic acid, which shows an IC_50_ of 3.7 μM and EC_max_ of >95% (Fierro et al., 2023). Whilst this suggests that the antagonist is highly effective at blocking receptor activity, molecular knockdown of T2R14, for example using specific siRNA, would be useful to confirm the protective effect of T2R14 in attenuating VEGF‐induced angiogenic processes. Activation of endothelial T2Rs has been previously shown to regulate intracellular signalling pathways involved in vascular function, including cAMP‐dependent pathways and modulation of endothelial junctional proteins, demonstrating that bitter taste receptors can influence endothelial phenotype (Kertesz et al., 2022). Since VEGF‐induced adhesion, migration and tube formation require coordinated activation of VEGFR2‐associated downstream pathways, including PI3K‐AKT, ERK, Rac1 and cytoskeletal remodelling, T2R14 antagonism may reduce angiogenic responses by altering GPCR‐mediated crosstalk with these pathways. Indeed, modulation of endothelial GPCR activity has previously been shown to suppress VEGF‐induced migration and tube formation by interfering with downstream Akt/eNOS signalling without necessarily affecting VEGFR activation itself (Benndorf et al., 2003). Interestingly, our studies also demonstrated that of all the angiogenesis regulators studied, only CXCL16, endoglin, FGF‐7, and VEGF‐C were affected by LW129 and VEGF co‐treatment. Indeed, all 4 were significantly downregulated, suggesting a combined or synergistic effect of the T2R14 antagonist with VEGF. A plausible model is that CXCL16, FGF‐7, VEGF‐C, and endoglin form complementary components of a pro‐angiogenic/remodelling network in the pulmonary vascular niche, although direct evidence demonstrating a single, communal pathway in the lung endothelium is currently limited. CXCL16 can directly stimulate endothelial migration, proliferation, and tube formation through ERK‐dependent signalling and can increase endothelial VEGF production via ERK/p38/AKT–HIF‐1α pathways, potentially sensitising the endothelium to subsequent growth‐factor stimulation (Yu et al., 2016; Zhuge et al., 2005). FGF‐7, in microvascular endothelial models, promotes chemotaxis, proliferation, and MAPK activation which could be accelerated by VEGF‐C through VEGFR‐2/VEGFR‐3 signalling (Cao et al., 1998; Gillis et al., 1999). Finally, endoglin, an endothelial TGF‐β co‐receptor, can interact with VEGFR2 to sustain receptor surface expression and enhance VEGF‐dependent endothelial sprouting (Tian et al., 2018). It is important to acknowledge that the findings in the present study were derived from a protein array. Although the array was prepared using specific antibodies, using a sandwich immunoassay approach, the observed changes in protein expression should be independently validated using complementary techniques, such as Western blotting or ELISA. Such validation would provide additional confirmation of the observed differences in protein expression and strengthen the conclusions drawn from these findings. However, it is worth noting that our studies with LW129 treatment alone demonstrate no significant change in VEGF‐C expression using both the Proteome Profiler™ array (Figure 4c iv) and ELISA (Figure 2e). The concordance between these two independent approaches provides support for the reliability of the array findings in this context. Nevertheless, it should be acknowledged that the Proteome Profiler™ provides relative rather than absolute measures of protein expression, and further validation of individual proteins using quantitative assays would strengthen these findings. It is therefore possible that LW129 exposure, in VEGF‐induced, pro‐angiogenic settings could blunt this complementary signalling cascade to block angiogenic remodelling of the lung microvasculature. Interestingly, VEGF‐C is the only studied protein that was significantly upregulated with VEGF exposure and significantly attenuated when LW129 was added in co‐treatment. These are the first studies to link T2R14 with VEGF‐C; therefore, there is limited mechanistic detail on how this association occurs. Given the established T2R14 signalling observed in other cell lines, it is possible that LW129 could reduce VEGF‐C transcription by shifting pathways such as PI3K‐Akt and MAPK/ERK to reduce the VEGF signal and block endothelial angiogenic processes (Kim et al., 2021; Sodhi et al., 2001). However, significant further investigation of how T2R14 could impact these signalling molecules, VEGF‐C specifically, is needed to better understand whether this pathway is directly responsible for the attenuation of angiogenic processes seen in the present study.

## 4 METHODS

### 4.1 Cell culture and reagents

T2R14 antagonist LW129 was designed based on Fierro et al (Fierro et al., 2023) and produced by Enamine (Germany). Recombinant human VEGF (PHC9394) was obtained from ThermoFisher (UK). Human Pulmonary Microvascular Endothelial Cells from male donors (HPMECs; CC‐2527), Endothelial Cell Growth Basal Medium‐2 (EBM™‐2; CC‐3156) and Endothelial Cell Growth Medium SingleQuots™ Supplements (EGM™‐2; CC‐4176) were obtained from Lonza (Switzerland). Phosphate buffered saline (PBS) and BCA protein assay kit was obtained from Severn Biotech (UK) and Thermo Scientific (UK), respectively. Gelatin (G2500), aristolochic acid (PHL89565), tangeretin (T8951), flufenamic acid (F9005), nobiletin (N1538) and luteolin (L9283) were purchased from Merck (UK). Proteome Profiler kits for Human Angiogenesis array (ARY007) and Matrigel™ (354234) were obtained from Bio‐Techne (UK) and Corning (UK), respectively. All other reagents were purchased from Sigma Aldrich, UK.

HPMECs were cultured on 1% gelatin and were maintained in endothelial cell growth medium supplemented with 2% fetal bovine serum (FBS), the SingleQuots™ Supplements and 1% penicillin–streptomycin. Cultures were incubated at 37°C in a humidified atmosphere containing 5% CO_2_. Medium was replaced every 2–3 days, and cells were passaged at ~80%–90% confluence using trypsin–EDTA. Experiments were performed using cells up to passage 6.

### 4.2 MTT viability assay

Cell viability was assessed using the MTT assay. HPMECs were seeded in 96‐well plates at a density of 2 × 10^4^ cells per well and allowed to adhere for 24 h. Cells were rinsed with PBS once and exposed to vehicle (DMSO 0.0005%), T2R14 agonists, aristolochic acid, flufenamic acid, luteolin and tangeretin, or the T2R14 antagonist, LW129, at concentrations ranging from 1 nM to 0.1 mM for a further 24 h. The cell viability assay was then performed using MTT solution prepared in PBS (5 mg/mL) followed by incubation for 3 h at 37°C. The supernatant was then carefully removed, and formazan crystals were dissolved using 10% SDS. Absorbance was measured at 570 nm using a microplate reader (Tecan Sunrise). Cell viability was expressed as a percentage relative to vehicle control. For each experiment, 3 technical repeats were performed and analysed per condition to give 1 biological replicate. 3 and 4 biological replicates were performed for T2R14 agonists and antagonist, respectively.

### 4.3 Cell proliferation and adhesion

Cell adhesion was quantified using the MTT assay. HPMECs were prepared as a pellet of 2 × 10^5^ cells per treatment and resuspended in media supplemented with LW129 (500 nM) or vehicle control (DMSO) in the presence or absence of VEGF (100 ng/mL) or VEGF vehicle control (H_2_O). Cell solutions were plated onto 96‐well wells for 6 h. Adhesion was then measured using the MTT assay as described in the *MTT viability* section. For the cell proliferation assay, HPMECs were plated in 24‐well plates at a density of 2 × 10^5^ cells per well. Following 24 h culture, cells were exposed to LW129 (500 nM) or vehicle control (DMSO) in the presence or absence of VEGF (100 ng/mL) or VEGF vehicle control (H_2_O) for a further 24 h. HPMEC were then collected using trypsin neutralised with media and counted using a haemocytometer with data presented as cells × 10^6^ per mL. For both adhesion and proliferation assays, 2 technical repeats were performed and analysed per condition to give 1 biological replicate. 3 biological replicates were performed.

### 4.4 Cell migration and tube formation assay

Cell migration was assessed using a wound healing (scratch) assay. HPMECs were seeded at a density of 5 × 10^4^ cells per well and grown to 80% confluence in 24‐well plates for 24 h. A linear scratch was created using a sterile pipette tip, and detached cells were removed by washing with PBS. Cells were then incubated in media supplemented with LW129 (500 nM) or vehicle control (DMSO) in the presence or absence of VEGF (100 ng/mL) or VEGF vehicle control (H_2_O) for 16 h. Images of the scratch were captured using the Zoe imaging microscope (BioRad). Cell migration was quantified using the Fiji ImageJ plugin ‘WoundHealingSizeToolUpdated’ and analysed as wound closure as a % of the original area. For each experiment, 2 technical repeats were performed and analysed per condition to give 1 biological replicate. 5 biological replicates were performed.

Angiogenic capacity was assessed using a Matrigel™ tube formation assay. Pre‐chilled 96‐well plates were coated with growth factor‐reduced Matrigel™ and incubated at 37°C for 30 min to allow polymerisation. HPMECs were seeded onto the Matrigel™ layer at a density of 5 × 10^4^ cells per well and incubated under experimental conditions. Tube formation was monitored and imaged after 20 h using a Zoe imaging microscope (BioRad). Quantitative analysis of branch length, number of segments, and number of branches was performed using the Fiji ImageJ plugin ’AngiogenesisAnalyzer’. For each experiment, 2 technical repeats were performed and analysed per condition to give 1 biological replicate. 6 biological replicates were performed.

### 4.5 Proteome profiler analysis

The Human Proteome Profiler kit specific to Human Angiogenesis (ARY007) was used as per manufacturer guidelines. In brief, HPMECs were exposed to LW129 (500 nM) or vehicle control (DMSO) in the presence and absence of VEGF (100 ng/mL) or vehicle control (H_2_O) for 24 h. Cell lysates were collected in RIPA lysis buffer on ice, incubated for 30 min at 4°C and then centrifuged at 14,000 × *g* for 15 min. Supernatants were collected and protein concentration was quantified using the BCA assay. The Proteome Profiler kit membranes were blocked for 1 h, followed by exposure to lysate and antibody mixture overnight at 4°C. Following 3 washes, membranes were exposed to streptavidin‐HRP for 30 min at room temperature, and then chemiluminescence reagent for 1 min. Blots were visualised using the IBright imaging system and analysed using the Fiji ImageJ plugin ‘ProteinArrayAnalyzer’. Preloaded templates for the kit were aligned with blot images, pixel density was corrected for background density, and percentage change in expression was calculated. For each experiment, 1 technical repeat was performed and analysed per condition to give 1 biological replicate. 3 biological replicates were performed.

### 4.6 Statistical analysis

All experiments were performed with at least 3 biological replicates. Data were analysed using unpaired two‐tailed Student's *t*‐test or one‐way ANOVA where appropriate, using GraphPad Prism (Version 7.05). For one‐way ANOVA testing, Brown‐Forsythe test and Bartlett's test were used to assess equality of group variances and homogeneity of variances. For one‐way ANOVA and two‐way ANOVA, Holm‐Sidak's and Tukey's multiple comparisons tests were used respectively. Significance was defined as *p* ≤ 0.05.

## AUTHOR CONTRIBUTIONS

**Lewis Spurrier‐Best:** conceptualization, data curation, formal analysis, investigation, methodology, writing – review and editing. **Ellizabeth O. Harrington:** conceptualization, supervision, validation, writing – review and editing. **Havovi Chichger:** conceptualization, formal analysis, project administration, resources, supervision, writing – original draft.

## FUNDING INFORMATION

This material is the result of work supported with resources and the use of facilities at Anglia Ruskin University. The work is supported by a Cambridge Philosophical Society research grant awarded to EOH and HC (CPS2023‐467403).

## CONFLICT OF INTEREST STATEMENT

The authors declare that they have no competing interests.

## ETHICS STATEMENT

Not applicable.

## Data Availability

The datasets generated and analysed during the current study are available from the corresponding author upon reasonable request.

## References

[phy271168-bib-0001] Abel, S. , Hundhausen, C. , Mentlein, R. , Schulte, A. , Berkhout, T. A. , Broadway, N. , Hartmann, D. , Sedlacek, R. , Dietrich, S. , Muetze, B. , Schuster, B. , Kallen, K. , Saftig, P. , Rose‐John, S. , & Ludwig, A. (2004). The transmembrane CXC‐chemokine ligand 16 is induced by IFN‐gamma and TNF‐alpha and shed by the activity of the disintegrin‐like metalloproteinase ADAM10. Journal of Immunology, 172(10), 6362–6372. 10.4049/jimmunol.172.10.6362 15128827

[phy271168-bib-0002] Abhinand, C. S. , Galipon, J. , Mori, M. , Ramesh, P. , Prasad, T. S. K. , Raju, R. , Sudhakaran, P. R. , & Tomita, M. (2023). Temporal phosphoproteomic analysis of VEGF‐A signaling in HUVECs: An insight into early signaling events associated with angiogenesis. Journal of Cell Communication and Signaling, 17(3), 1067–1079. 10.1007/s12079-023-00736-z PMC10409921 36881336

[phy271168-bib-0003] Ackermann, M. , Werlein, C. , Plucinski, E. , Leypold, S. , Kuhnel, M. P. , Verleden, S. E. , Khalil, H. A. , Langer, F. , Welte, T. , Mentzer, S. J. , & Jonigk, D. D. (2024). The role of vasculature and angiogenesis in respiratory diseases. Angiogenesis, 27(3), 293–310. 10.1007/s10456-024-09910-2 PMC11303512 38580869

[phy271168-bib-0004] Battegay, E. J. , Rupp, J. , Iruela‐Arispe, L. , Sage, E. H. , & Pech, M. (1994). PDGF‐BB modulates endothelial proliferation and angiogenesis in vitro via PDGF beta‐receptors. The Journal of Cell Biology, 125(4), 917–928. 10.1083/jcb.125.4.917 PMC2120083 7514607

[phy271168-bib-0005] Becker, P. M. , Verin, A. D. , Booth, M. A. , Liu, F. , Birukova, A. , & Garcia, J. G. (2001). Differential regulation of diverse physiological responses to VEGF in pulmonary endothelial cells. American Journal of Physiology. Lung Cellular and Molecular Physiology, 281(6), 1500–L1511. 10.1152/ajplung.2001.281.6.L1500 11704547

[phy271168-bib-0006] Benndorf, R. , Boger, R. H. , Ergun, S. , Steenpass, A. , & Wieland, T. (2003). Angiotensin II type 2 receptor inhibits vascular endothelial growth factor‐induced migration and in vitro tube formation of human endothelial cells. Circulation Research, 93(5), 438–447. 10.1161/01.RES.0000088358.99466.04 12881481

[phy271168-bib-0007] Cao, Y. , Linden, P. , Farnebo, J. , Cao, R. , Eriksson, A. , Kumar, V. , Qi, J. H. , Claesson‐Welsh, L. , & Alitalo, K. (1998). Vascular endothelial growth factor C induces angiogenesis in vivo. Proceedings of the National Academy of Sciences of the United States of America, 95(24), 14389–14394. 10.1073/pnas.95.24.14389 PMC24383 9826710

[phy271168-bib-0008] Cao, Z. , Lis, R. , Ginsberg, M. , Chavez, D. , Shido, K. , Rabbany, S. Y. , Fong, G. , Sakmar, T. P. , Rafii, S. , & Ding, B. (2016). Targeting of the pulmonary capillary vascular niche promotes lung alveolar repair and ameliorates fibrosis. Nature Medicine, 22(2), 154–162. 10.1038/nm.4035 PMC4872630 26779814

[phy271168-bib-0009] Carey, R. M. , Workman, A. D. , Yan, C. H. , Chen, B. , Adappa, N. D. , Palmer, J. N. , Kennedy, D. W. , Lee, R. J. , & Cohen, N. A. (2017). Sinonasal T2R‐mediated nitric oxide production in response to Bacillus cereus. American Journal of Rhinology & Allergy, 31(4), 211–215. 10.2500/ajra.2017.31.4453 PMC5498317 28716170

[phy271168-bib-0010] Chandrashekar, J. , Mueller, K. L. , Hoon, M. A. , Adler, E. , Feng, L. , Guo, W. , Zuker, C. S. , & Ryba, N. J. (2000). T2Rs function as bitter taste receptors. Cell, 100(6), 703–711. 10.1016/s0092-8674(00)80706-0 10761935

[phy271168-bib-0011] Di Pizio, A. , Waterloo, L. A. W. , Brox, R. , Lober, S. , Weikert, D. , Behrens, M. , Gmeiner, P. , & Niv, M. Y. (2020). Rational design of agonists for bitter taste receptor TAS2R14: From modeling to bench and back. Cellular and Molecular Life Sciences, 77(3), 531–542. 10.1007/s00018-019-03194-2 PMC11104859 31236627

[phy271168-bib-0012] Dudley, A. C. , & Griffioen, A. W. (2023). Pathological angiogenesis: Mechanisms and therapeutic strategies. Angiogenesis, 26(3), 313–347. 10.1007/s10456-023-09876-7 PMC10105163 37060495

[phy271168-bib-0013] Eelen, G. , Treps, L. , Li, X. , & Carmeliet, P. (2020). Basic and therapeutic aspects of angiogenesis updated. Circulation Research, 127(2), 310–329. 10.1161/CIRCRESAHA.120.316851 32833569

[phy271168-bib-0014] Fierro, F. , Peri, L. , Hubner, H. , Tabor‐Schkade, A. , Waterloo, L. , Lober, S. , Pfeiffer, T. , Weikert, D. , Dingjan, T. , Margulis, E. , Gmeiner, P. , & Niv, M. Y. (2023). Inhibiting a promiscuous GPCR: Iterative discovery of bitter taste receptor ligands. Cellular and Molecular Life Sciences, 80(4), 114. 10.1007/s00018-023-04765-0 PMC11072104 37012410

[phy271168-bib-0015] Foster, S. R. , Porrello, E. R. , Purdue, B. , Chan, H. , Voigt, A. , Frenzel, S. , Hannan, R. D. , Moritz, K. M. , Simmons, D. G. , Molenaar, P. , Roura, E. , Boehm, U. , Meyerhof, W. , & Thomas, W. G. (2013). Expression, regulation and putative nutrient‐sensing function of taste GPCRs in the heart. PLoS One, 8(5), e64579. 10.1371/journal.pone.0064579 PMC3655793 23696900

[phy271168-bib-0016] Fujio, Y. , & Walsh, K. (1999). Akt mediates cytoprotection of endothelial cells by vascular endothelial growth factor in an anchorage‐dependent manner. The Journal of Biological Chemistry, 274(23), 16349–16354. 10.1074/jbc.274.23.16349 PMC3624707 10347193

[phy271168-bib-0017] Gerber, H. P. , McMurtrey, A. , Kowalski, J. , Yan, M. , Keyt, B. A. , Dixit, V. , & Ferrara, N. (1998). Vascular endothelial growth factor regulates endothelial cell survival through the phosphatidylinositol 3′‐kinase/Akt signal transduction pathway. Requirement for Flk‐1/KDR activation. Journal of Biological Chemistry, 273(46), 30336–30343. 10.1074/jbc.273.46.30336 9804796

[phy271168-bib-0018] Ghofrani, H. , Gomberg‐Maitland, M. , Zhao, L. , & Grimminger, F. (2025). Mechanisms and treatment of pulmonary arterial hypertension. Nature Reviews Cardiology, 22(2), 105–120. 10.1038/s41569-024-01064-4 39112561

[phy271168-bib-0019] Gillis, P. , Savla, U. , Volpert, O. V. , Jimenez, B. , Waters, C. M. , Panos, R. J. , & Bouck, N. P. (1999). Keratinocyte growth factor induces angiogenesis and protects endothelial barrier function. Journal of Cell Science, 112(12), 2049–2057. 10.1242/jcs.112.12.2049 10341222

[phy271168-bib-0020] Isozaki, T. , Arbab, A. S. , Haas, C. S. , Amin, M. A. , Arendt, M. D. , Koch, A. E. , & Ruth, J. H. (2013). Evidence that CXCL16 is a potent mediator of angiogenesis and is involved in endothelial progenitor cell chemotaxis: Studies in mice with K/BxN serum‐induced arthritis. Arthritis and Rheumatism, 65(7), 1736–1746. 10.1002/art.37981 PMC3701743 23633118

[phy271168-bib-0021] Karaman, R. , Nowak, S. , Di Pizio, A. , Kitaneh, H. , Abu‐Jaish, A. , Meyerhof, W. , Niv, M. Y. , & Behrens, M. (2016). Probing the binding pocket of the broadly tuned human bitter taste receptor TAS2R14 by chemical modification of cognate agonists. Chemical Biology & Drug Design, 88(1), 66–75. 10.1111/cbdd.12734 26825540

[phy271168-bib-0022] Kertesz, Z. , Harrington, E. O. , Braza, J. , Guarino, B. D. , & Chichger, H. (2022). Agonists for bitter taste receptors T2R10 and T2R38 attenuate LPS‐induced permeability of the pulmonary endothelium in vitro. Frontiers in Physiology, 13, 794370. 10.3389/fphys.2022.794370 PMC8985831 35399266

[phy271168-bib-0023] Kim, D. , Castano, M. , Lujan, L. K. , Woo, J. A. , & Liggett, S. B. (2021). The short third intracellular loop and cytoplasmic tail of bitter taste receptors provide functionally relevant GRK phosphorylation sites in TAS2R14. The Journal of Biological Chemistry, 296, 100216. 10.1074/jbc.RA120.016056 PMC7949105 33465377

[phy271168-bib-0024] Kim, Y. M. , Song, E. J. , Seo, J. , Kim, H. , & Lee, K. (2007). Proteomic analysis of tyrosine phosphorylations in vascular endothelial growth factor‐ and reactive oxygen species‐mediated signaling pathway. Journal of Proteome Research, 6(2), 593–601. 10.1021/pr060326s 17269716

[phy271168-bib-0025] Levit, A. , Nowak, S. , Peters, M. , Wiener, A. , Meyerhof, W. , Behrens, M. , & Niv, M. Y. (2014). The bitter pill: Clinical drugs that activate the human bitter taste receptor TAS2R14. The FASEB Journal, 28(3), 1181–1197. 10.1096/fj.13-242594 24285091

[phy271168-bib-0026] Lu, Y. , Liang, L. , Cheng, P. , Xiong, L. , Wang, M. , Song, L. , Yu, F. , He, X. , Xiong, L. , Wang, X. , Xin, J. , Ye, H. , & Ma, W. (2021). VEGF/Src signaling mediated pleural barrier damage and increased permeability contributes to subpleural pulmonary fibrosis. American Journal of Physiology. Lung Cellular and Molecular Physiology, 320(6), L990–L1004. 10.1152/ajplung.00436.2020 33787325

[phy271168-bib-0027] McMahon, D. B. , Kuek, L. E. , Johnson, M. E. , Johnson, P. O. , Horn, R. L. J. , Carey, R. M. , Adappa, N. D. , Palmer, J. N. , & Lee, R. J. (2022). The bitter end: T2R bitter receptor agonists elevate nuclear calcium and induce apoptosis in non‐ciliated airway epithelial cells. Cell Calcium, 101, 102499. 10.1016/j.ceca.2021.102499 PMC8752513 34839223

[phy271168-bib-0028] Mirza, S. L. , Upton, P. D. , Hodgson, J. , Graf, S. , Morrell, N. W. , & Dunmore, B. J. (2024). SEMA3G regulates BMP9 inhibition of VEGF‐mediated migration and network formation in pulmonary endothelial cells. Vascular Pharmacology, 155, 107381. 10.1016/j.vph.2024.107381 38795838

[phy271168-bib-0029] Mocumbi, A. , Humbert, M. , Saxena, A. , Jing, Z. , Sliwa, K. , Thienemann, F. , Archer, S. L. , & Stewart, S. (2024). Pulmonary hypertension. Nature Reviews. Disease Primers, 10(1), 1–7. 10.1038/s41572-023-00486-7 38177157

[phy271168-bib-0030] Nowak, S. , Di Pizio, A. , Levit, A. , Niv, M. Y. , Meyerhof, W. , & Behrens, M. (2018). Reengineering the ligand sensitivity of the broadly tuned human bitter taste receptor TAS2R14. Biochimica et Biophysica Acta, General Subjects, 1862(10), 2162–2173. 10.1016/j.bbagen.2018.07.009 30009876

[phy271168-bib-0031] Ourradi, K. , Blythe, T. , Jarrett, C. , Barratt, S. L. , Welsh, G. I. , & Millar, A. B. (2017). VEGF isoforms have differential effects on permeability of human pulmonary microvascular endothelial cells. Respiratory Research, 18(1), 116. 10.1186/s12931-017-0602-1 PMC5457598 28578669

[phy271168-bib-0032] Papaioannou, A. I. , Kostikas, K. , Kollia, P. , & Gourgoulianis, K. I. (2006). Clinical implications for vascular endothelial growth factor in the lung: Friend or foe? Respiratory Research, 7(1), 128. 10.1186/1465-9921-7-128 PMC1629021 17044926

[phy271168-bib-0033] Perez‐Gutierrez, L. , & Ferrara, N. (2023). Biology and therapeutic targeting of vascular endothelial growth factor a. Nature Reviews. Molecular Cell Biology, 24(11), 816–834. 10.1038/s41580-023-00631-w 37491579

[phy271168-bib-0034] Robinett, K. S. , Koziol‐White, C. J. , Akoluk, A. , An, S. S. , Panettieri, R. A. J. , & Liggett, S. B. (2014). Bitter taste receptor function in asthmatic and nonasthmatic human airway smooth muscle cells. American Journal of Respiratory Cell and Molecular Biology, 50(4), 678–683. 10.1165/rcmb.2013-0439RC PMC4068928 24219573

[phy271168-bib-0035] Shibuya, M. (2011). Vascular endothelial growth factor (VEGF) and its receptor (VEGFR) signaling in angiogenesis: A crucial target for anti‐ and pro‐angiogenic therapies. Genes & Cancer, 2(12), 1097–1105. 10.1177/1947601911423031 PMC3411125 22866201

[phy271168-bib-0036] Sodhi, A. , Montaner, S. , Miyazaki, H. , & Gutkind, J. S. (2001). MAPK and Akt act cooperatively but independently on hypoxia inducible factor‐1alpha in rasV12 upregulation of VEGF. Biochemical and Biophysical Research Communications, 287(1), 292–300. 10.1006/bbrc.2001.5532 11549290

[phy271168-bib-0037] Song, M. , & Finley, S. D. (2022). Mechanistic characterization of endothelial sprouting mediated by pro‐angiogenic signaling. Microcirculation, 29: 2, e12744. 10.1111/micc.12744 PMC9285777 34890488

[phy271168-bib-0038] Stevens, R. P. , Alexeyev, M. F. , Kozhukhar, N. , Pastukh, V. , Paudel, S. S. , Bell, J. , Tambe, D. T. , Stevens, T. , & Lee, J. Y. (2022). Carbonic anhydrase IX proteoglycan‐like and intracellular domains mediate pulmonary microvascular endothelial cell repair and angiogenesis. American Journal of Physiology. Lung Cellular and Molecular Physiology, 323(1), L48–L57. 10.1152/ajplung.00337.2021 PMC9255709 35672011

[phy271168-bib-0039] Sun, Z. , Li, X. , Massena, S. , Kutschera, S. , Padhan, N. , Gualandi, L. , Sundvold‐Gjerstad, V. , Gustafsson, K. , Choy, W. W. , Zang, G. , Quach, M. , Jansson, L. , Phillipson, M. , Abid, M. R. , Spurkland, A. , & Claesson‐Welsh, L. (2012). VEGFR2 induces c‐Src signaling and vascular permeability in vivo via the adaptor protein TSAd. The Journal of Experimental Medicine, 209(7), 1363–1377. 10.1084/jem.20111343 PMC3405501 22689825

[phy271168-bib-0040] Thommen, R. , Humar, R. , Misevic, G. , Pepper, M. S. , Hahn, A. W. , John, M. , & Battegay, E. J. (1997). PDGF‐BB increases endothelial migration on cord movements during angiogenesis in vitro. Journal of Cellular Biochemistry, 64(3), 403–413. 9057098

[phy271168-bib-0041] Tian, H. , Huang, J. J. , Golzio, C. , Gao, X. , Hector‐Greene, M. , Katsanis, N. , & Blobe, G. C. (2018). Endoglin interacts with VEGFR2 to promote angiogenesis. The FASEB Journal, 32(6), 2934–2949. 10.1096/fj.201700867RR 29401587

[phy271168-bib-0042] Walsh, D. , Kostyunina, D. S. , Blake, A. , Boylan, J. , & McLoughlin, P. (2025). Shear stress‐induced restoration of pulmonary microvascular endothelial barrier function following ischemia reperfusion injury requires VEGFR2 signaling. American Journal of Physiology. Lung Cellular and Molecular Physiology, 328(3), L389–L404. 10.1152/ajplung.00200.2024 39701597

[phy271168-bib-0043] Yu, X. , Zhao, R. , Lin, S. , Bai, X. , Zhang, L. , Yuan, S. , & Sun, L. (2016). CXCL16 induces angiogenesis in autocrine signaling pathway involving hypoxia‐inducible factor 1alpha in human umbilical vein endothelial cells. Oncology Reports, 35(3), 1557–1565. 10.3892/or.2015.4520 26707275

[phy271168-bib-0044] Zhuge, X. , Murayama, T. , Arai, H. , Yamauchi, R. , Tanaka, M. , Shimaoka, T. , Yonehara, S. , Kume, N. , Yokode, M. , & Kita, T. (2005). CXCL16 is a novel angiogenic factor for human umbilical vein endothelial cells. Biochemical and Biophysical Research Communications, 331(4), 1295–1300. 10.1016/j.bbrc.2005.03.200 15883016

